# Efficacy of Integrating the Management of Pain and Addiction via Collaborative Treatment (IMPACT) in Individuals With Chronic Pain and Opioid Use Disorder: Protocol for a Randomized Clinical Trial of a Digital Cognitive Behavioral Treatment

**DOI:** 10.2196/54342

**Published:** 2024-03-20

**Authors:** R Ross MacLean, Brett Ankawi, Mary A Driscoll, Melissa A Gordon, Tami L Frankforter, Charla Nich, Sara K Szollosy, Jennifer M Loya, Larissa Brito, Margaridha I P Ribeiro, Sara N Edmond, William C Becker, Steve Martino, Mehmet Sofuoglu, Alicia A Heapy

**Affiliations:** 1 VA Connecticut Healthcare System West Haven, CT United States; 2 School of Medicine Yale University New Haven, CT United States; 3 Pain Research, Informatics, Multimorbidities, and Education (PRIME) Health Services Research and Development Center of Innovation VA Connecticut Healthcare System West Haven, CT United States

**Keywords:** chronic pain, digital treatment, medications for opioid use disorder, methadone, opioid use disorder

## Abstract

**Background:**

Chronic pain is common among individuals with opioid use disorder (OUD) who are maintained on medications for OUD (MOUD; eg, buprenorphine or methadone). Chronic pain is associated with worse retention and higher levels of substance use. Treatment of individuals with chronic pain receiving MOUD can be challenging due to their increased clinical complexity. Given the acute and growing nature of the opioid crisis, MOUD is increasingly offered in a wide range of settings, where high-quality, clinician-delivered, empirically validated behavioral treatment for chronic pain may not be available. Therefore, digital treatments that support patient self-management of chronic pain and OUD have the potential for wider implementation to fill this gap.

**Objective:**

This study aims to evaluate the efficacy of Integrating the Management of Pain and Addiction via Collaborative Treatment (IMPACT), an interactive digital treatment program with asynchronous coach feedback, compared to treatment as usual (TAU) in individuals with chronic pain and OUD receiving MOUD.

**Methods:**

Adult participants (n=160) receiving MOUD and reporting bothersome or high-impact chronic pain will be recruited from outpatient opioid treatment programs in Connecticut (United States) and randomized 1:1 to either IMPACT+TAU or TAU only. Participants randomized to IMPACT+TAU will complete an interactive digital treatment that includes 9 modules promoting training in pain and addiction coping skills and a progressive walking program. The program is augmented with a weekly personalized voice message from a trained coach based on daily participant-reported pain intensity and interference, craving to use opioids, sleep quality, daily steps, pain self-efficacy, MOUD adherence, and engagement with IMPACT collected through digital surveys. Outcomes will be assessed at 3, 6, and 9 months post randomization. The primary outcome is MOUD retention at 3 months post randomization (ie, post treatment). Secondary outcomes include pain interference, physical functioning, MOUD adherence, substance use, craving, pain intensity, sleep disturbance, pain catastrophizing, and pain self-efficacy. Semistructured qualitative interviews with study participants (n=34) randomized to IMPACT (completers and noncompleters) will be conducted to evaluate the usability and quality of the program and its outcomes.

**Results:**

The study has received institutional review board approval and began recruitment at 1 site in July 2022. Recruitment at a second site started in January 2023, with a third and final site anticipated to begin recruitment in January 2024. Data collection is expected to continue through June 2025.

**Conclusions:**

Establishing efficacy for a digital treatment for addiction and chronic pain that can be integrated into MOUD clinics will provide options for individuals with OUD, which reduce barriers to behavioral treatment. Participant feedback on the intervention will inform updates or modifications to improve engagement and efficacy.

**Trial Registration:**

ClinicalTrials.gov NCT05204576; https://clinicaltrials.gov/ct2/show/NCT05204576

**International Registered Report Identifier (IRRID):**

DERR1-10.2196/54342

## Introduction

### Background

Despite increased attention from the media, policy makers, researchers, and clinicians, the opioid epidemic in the United States remains a significant public health crisis. Approximately 107,000 US adults died by drug overdose in 2021, with increases in every age group and an overall age-adjusted 14% increase from the previous year [1]. An estimated two-thirds of overdose deaths involved synthetic opioids other than methadone (eg, illicitly manufactured fentanyl) [2]. Medications for opioid use disorder (MOUD), including buprenorphine and methadone, are protective against mortality in individuals with opioid use disorder (OUD) [3,4], reducing overdose death and all-cause death by 8 and 2.5 times, respectively [5]. Furthermore, longer retention in MOUD is associated with a continued reduction in mortality [5]. Retention of at least 6 months, and often longer, is needed to obtain full benefits, including a return to work and improvements in fulfilling family and social responsibilities [6,7]. Despite these benefits, only 36% of individuals who initiate are retained in MOUD for 6 months, with retention rates falling to 22% at 1 year [8]. Therefore, developing interventions that support MOUD retention and address co-occurring conditions that interfere with retention is essential to enabling people with OUD to obtain the full benefits of MOUD treatment.

### Role of Pain in the Development of OUD

One condition that commonly co-occurs with OUD is chronic pain, frequently defined as pain that persists for 3 months or more. Chronic pain is a widespread affliction in the United States, affecting approximately 1 in 5 adults [9], and is more common among people with OUD. A large study of electronic health records revealed that 64.4% of individuals with OUD also had a chronic pain condition [10]. Chronic pain can be a pathway to OUD, with 4 out of 5 individuals who use heroin reporting their initial exposure to opioids was through a prescription for pain treatment [11,12]. It is a common misconception, even among addiction providers, that methadone and buprenorphine provide adequate pain treatment for individuals with OUD [13]. Unsurprisingly, chronic pain typically persists when MOUD is the sole treatment modality. Among individuals enrolled in methadone or buprenorphine treatment, estimates of chronic pain range from 40% to 80% [14-17].

### Chronic Pain Negatively Affects MOUD Outcomes

The presence of chronic pain may undermine the effectiveness of MOUD. A large randomized, controlled multisite trial of buprenorphine in individuals with OUD found greater pain severity significantly increased the odds of opioid use in the following week [18]. Follow-up analyses revealed that variability or volatility in pain intensity was associated with craving, relapse to opioid use, and poorer outcomes [18,19]. Furthermore, the experience of pain has been cited as the most common reason for returning to use while engaged in MOUD [20]. Finally, few people receiving MOUD receive adequate evidence-based pain care; this has been recognized as an important gap in the treatment of comorbid OUD and chronic pain [15,21-24].

Individuals with chronic pain receiving MOUD, compared to those without chronic pain, have higher rates of psychiatric and medical comorbidities, including sleep disturbances, and higher rates of health service use and levels of functional impairment [21,25-27]. Comprehensive treatment for individuals with concurrent chronic pain and OUD may require interventions that address mood, anxiety, sleep, and functional difficulties to maximize OUD treatment outcomes, such as MOUD retention. That is, chronic pain with OUD does not necessarily indicate more severe OUD; rather, it represents a concurrent disorder that may have a different clinical course and treatment response than OUD alone [22,28]. Accordingly, treatments should consider the relationship between chronic pain and opioid use while addressing barriers to engagement in resource-limited MOUD clinics.

### Cognitive Behavioral Therapy Is an Evidence-Based Treatment for Both Chronic Pain and Substance Use, but it has Limited Accessibility

Cognitive behavioral therapy (CBT) has a strong evidence base for the treatment of chronic pain [29] and substance use disorders [30-33]. CBT for chronic pain has been recommended by the Centers for Disease Control and Prevention and the National Institutes of Health (NIH) as a first-line treatment to reduce pain and improve function [34,35]. Although MOUD clinicians report interest in nonpharmacologic pain treatment, very few report confidence in their own ability to address chronic pain [36]. Similar to chronic pain, CBT for substance use disorders has been shown to have moderate effects on reducing substance use, promoting abstinence from substances, increasing the use of coping skills, and promoting other positive psychosocial outcomes in individuals with addiction [37]. Engagement in CBT for substance use disorder is a mainstay in outpatient and inpatient treatment, but, akin to CBT for chronic pain, implementing CBT with high fidelity is challenging due to the lack of trained clinicians in the setting [38,39]. For these reasons, offering adjunctive CBT in MOUD clinics has resulted in mixed findings [40-42]. Taken together, there is a critical gap in treatment where individuals with OUD receiving MOUD have few options for addiction or pain treatment. Accordingly, there has been a recent call to develop rigorous, evidence-based digital treatments for individuals with OUD, which can be integrated into MOUD clinical settings [43].

### Development of Integrating the Management of Pain and Addiction via Collaborative Treatment

**Figure 1:**
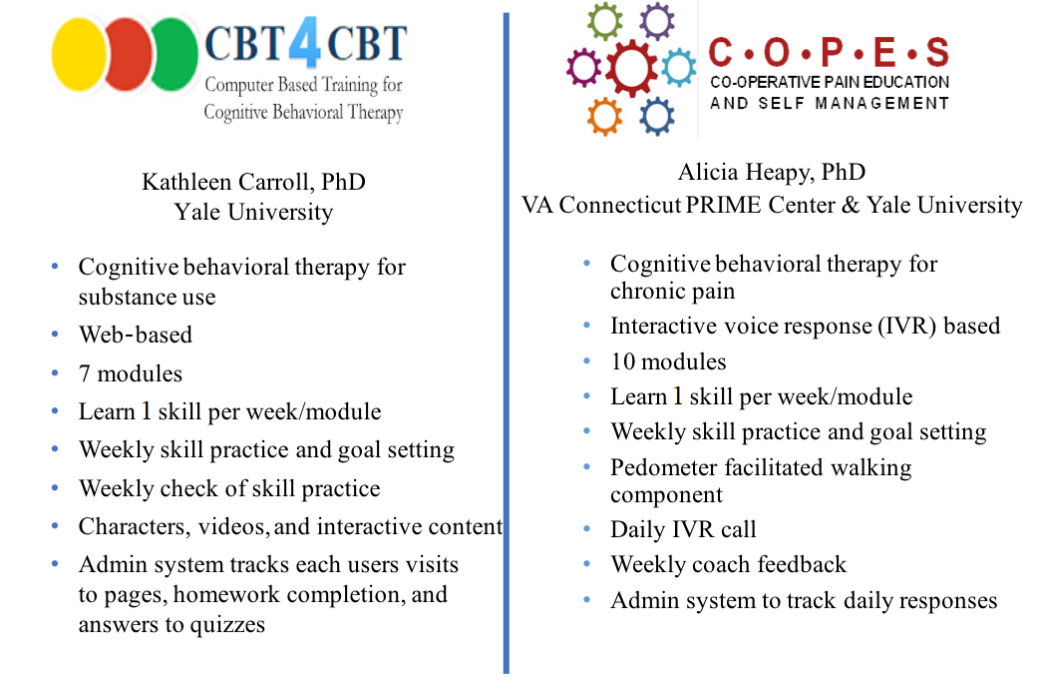
Core program features of the Computer-Based Treatment for Cognitive Behavioral Therapy (CBT4CBT) and Cooperative Pain Education and Self-Management (COPES) programs.

Integrating the Management of Pain and Addiction via Collaborative Treatment (IMPACT) combines 2 evidence-based digital CBT treatments: Computer-Based Treatment for Cognitive Behavioral Therapy (CBT4CBT) and Cooperative Pain Education and Self-Management (COPES) treatment programs (Figure 1). CBT4CBT is a web-based program designed to improve behavioral and cognitive coping skills that have been evaluated in a range of substance use disorders [44-47], including 2 trials indicating its effectiveness and durability for individuals with OUD on methadone maintenance [48,49] and office-based buprenorphine maintenance [50]. COPES is an interactive voice response–based treatment for chronic pain that is delivered asynchronously through telephone [51] and has been shown to be similarly effective in reducing pain intensity and improving physical functioning, sleep quality, and quality of life as real-time CBT with a therapist [52]. IMPACT was designed to provide an easily accessible, standardized evidence-based intervention for those with OUD and chronic pain that could ultimately be easily integrated into MOUD clinic settings.

This study was funded by the National Center for Complementary and Integrative Health’s Behavioral Research to Improve Medication-Based Treatment (BRIM) program. The goal of BRIM, funded by NIH’s Helping to End Addiction Long-Term (HEAL) initiative, is to examine the role of behavioral interventions in improving the outcomes of MOUD, particularly MOUD access and retention. The R61/R33 award described here consists of 2 phases: a preparatory R61 phase used to develop the intervention and identify recruiting sites and the R33 clinical trial that is the focus of this protocol.

### Study Objectives

The study is designed to evaluate the efficacy of IMPACT along with treatment as usual (IMPACT+TAU) versus TAU only at 3 months post randomization (primary end point). The primary OUD outcome is verified retention in MOUD treatment, defined as enrollment in MOUD (ie, yes or no), with evidence of MOUD use in the week before the 3-month time point. Secondary treatment outcomes will include pain interference, physical functioning, MOUD adherence, substance use, craving, pain intensity, sleep disturbance, pain catastrophizing, and pain self-efficacy at 3 months post randomization. The secondary aim is to evaluate the durability of effects for MOUD retention at 6 and 9 months post randomization. The tertiary aim is to examine treatment usability and quality through qualitative interviews with individuals randomized to IMPACT.

## Methods

### Study Design

**Figure 2:**
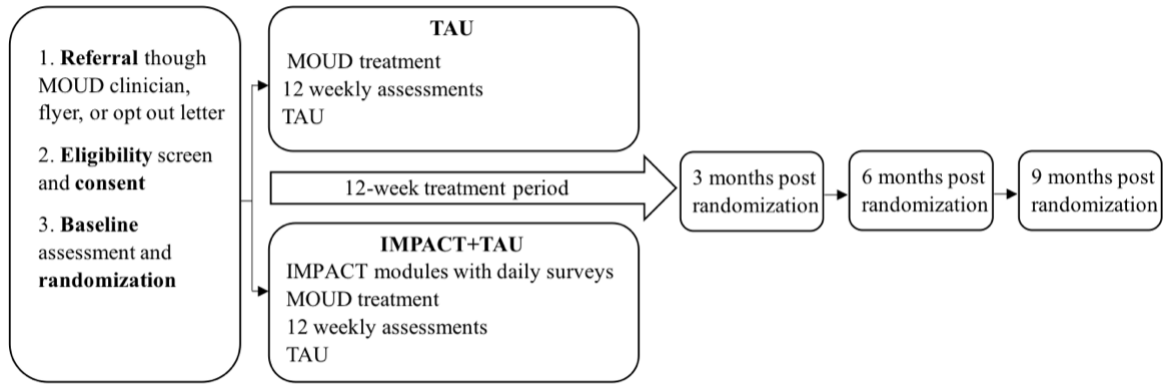
Study protocol for Integrating the Management of Pain and Addiction via Collaborative Treatment (IMPACT). MOUD: medications for opioid use disorder; TAU: treatment as usual.

This is a randomized clinical trial comparing the efficacy of IMPACT+TAU relative to TAU among individuals enrolled in MOUD treatment who have chronic pain. Outcomes will be assessed at baseline, 3, 6, and 9 months post randomization (Figure 2).

### Study Population and Setting

Participants will be 160 individuals with chronic pain receiving MOUD (methadone or buprenorphine) at participating outpatient opioid treatment program (OTP) sites located in Bridgeport, Danbury, and Waterbury (Connecticut).

### Ethical Considerations

The study was approved by the Yale University Institutional Review Board on September 4, 2019 (2000026276). Subsequent modifications were implemented to add posttreatment qualitative interviews to assess treatment satisfaction and usability, as well as to refine recruitment materials and add recruitment sites. The study was subsequently approved by the Veterans Affairs Connecticut Human Subjects Subcommittee because, although not enrolling Veteran Affairs patients, several study staff are employees of the Department of Veterans Affairs.

### Study Population

Textbox 1 contains inclusion and exclusion criteria for patients.

**Eligibility criteria for patients.**

**Inclusion criteria**

- Be 18 years of age or older
- Meet the
  Diagnostic and Statistical Manual of Mental Disorders (Fifth Edition) criteria for opioid use disorder and be enrolled in methadone or buprenorphine treatment at the participating medications for opioid use disorder (MOUD) clinic
- Receiving a stable (ie, unchanged in 2 weeks) dose of MOUD as judged by the prescribing clinician
- Report bothersome or high-impact chronic pain defined by the Graded Chronic Pain Scale–Revised
- Have a self-reported ability to walk at least 1 block

**Exclusion criteria**

- Inability to read, write, and speak English at a third-grade level
- Untreated or inadequately treated bipolar or psychotic disorder or current suicide risk for the previous 2 weeks before screening (identified using baseline measures)
- Life-threatening health conditions that would impede participation (eg, end-stage renal failure, malignant cancer requiring chemotherapy)
- Planned surgical treatment related to pain
- Pending legal action or planned relocation that would interfere with participation

### Justification for Criteria

Participants will be adults receiving a stable dose of MOUD because, until the dose is stable, pain ratings may fluctuate and obscure the intervention’s effect. We require bothersome or high-impact chronic pain (ie, moderate to severe pain that limits life and work activities on most days or every day in the past 3 months) because, compared to individuals with mild chronic pain, it is more commonly associated with negative health indicators addressed in cognitive behavioral treatment for chronic pain (eg, negative pain coping beliefs, pain interference, and psychological distress) [53]. IMPACT contains a progressive walking program; therefore, to ensure participant safety, participants must be able to walk at least 1 block. Individuals with low literacy (ie, <third grade) or medical or psychiatric diagnoses that would interfere with their ability to meaningfully participate in the IMPACT treatment, such as severe mental health conditions (eg, unmedicated or untreated bipolar or psychotic disorder, current suicide risk, or diagnoses requiring palliative or end-of-life care), are excluded. Individuals with circumstances that may predictably interfere with participation (eg, legal actions with imminent incarceration) or produce large changes in pain (pain-related surgeries) will be excluded. Women of childbearing age will be included, as there is no contraindication for CBT for pain or substance use disorder for pregnant women. Participants who do not have a digital device (eg, smartphone or tablet) for participating in IMPACT will be supplied with one for the duration of the study.

### Recruitment, Screening, and Consent Procedures

Study recruitment occurs through clinician referral of interested patients and patient self-referral in response to informational study materials posted in patient care areas at the recruiting sites. Site clinicians receive information about the study and eligibility criteria through research staff presentations and study informational material provided at clinical staff meetings. Self- and clinician-referred individuals are screened by a study research assistant and, if eligible, invited to consent and complete a baseline assessment. Procedures are conducted in a quiet and private office space provided by the OTP clinic to the research team. The research assistant and the participant discuss the consent document, and potential participants are provided an opportunity to ask questions and time to consider their decision to participate. A comprehension quiz is given to ensure the participant has an adequate understanding of the study, and a copy of the consent form is given to the participant. After consent, the research assistant assigns a unique study ID code and administers baseline measures on a study laptop using the REDCap (Research Electronic Data Capture) software platform.

### Randomization and Masking

Following the baseline assessment, participants are randomized 1:1 to IMPACT+TAU or TAU (control) using urn randomization [54] balanced by demographic variables (self-reported gender identification and race or ethnicity [combination of ethnicity and racial identity]), as well as likely prognostic variables (severity of OUD as mild, moderate, or severe based on the DSM-5 [*Diagnostic and Statistical Manual of Mental Disorders* {Fifth Edition} OUD symptom count), buprenorphine versus methadone treatment, and length of MOUD treatment (<6 months, 6-12 months, and >12 months). Allocation is masked using a Microsoft Access (Microsoft Corp) program that our group has developed and implemented successfully in multiple previous trials [55-58]. Participants randomized to IMPACT+TAU are provided with a unique username and password to access the IMPACT website. To facilitate their participation in the walking portion of the treatment, participants are given a pedometer and a brief demonstration of its use. Study staff measure each participant’s stride length and calibrate the pedometer accordingly. The research assistant then guides the participants through the first IMPACT module to ensure they are comfortable using the program and answer any questions they might have.

### Interventions

#### IMPACT

**Figure 3:**
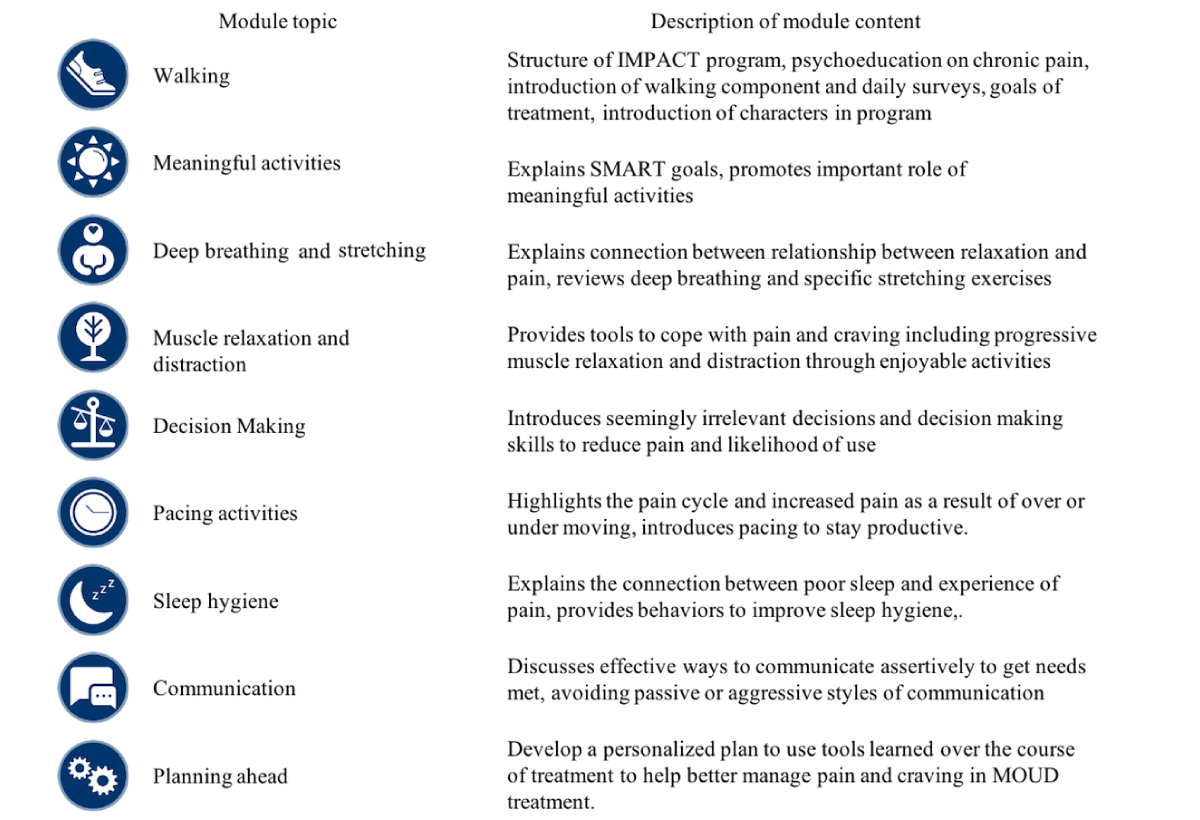
Integrating the Management of Pain and Addiction via Collaborative Treatment (IMPACT) module topics and descriptions. MOUD: medications for opioid use disorder; SMART: specific, measurable, achievable, relevant, and time-bound.

Participants allocated to IMPACT+TAU receive standard MOUD treatment (ie, TAU) along with access to IMPACT for 12 weeks. IMPACT is a self-directed, 9-module digital treatment program with weekly personalized feedback messages from a trained masters- or doctoral-level coach under the supervision of a clinical psychologist. Participants are asked to complete 1 module per week using a study laptop that is available in the MOUD clinic or outside of the clinic using their own web-enabled device (eg, at home). We hope to maximize treatment adherence and engagement by providing multiple ways of accessing the IMPACT program [50]. The IMPACT program passively tracks individual behaviors to capture engagement (ie, number of logins, access to each module and component, time spent using the program, and digital survey completion). Module topics are shown in Figure 3. For each module, participants are asked to practice a specific skill that corresponds with the topic for that week. After indicating a baseline average number of steps during their first week of completing the daily survey, participants are provided with a daily step goal by their coach during the weekly feedback message. The goal is to increase their daily steps by 10% of the average daily step count reported in the previous week. All modules include a narrated introduction of the skill, video vignettes of characters in challenging situations and using skills, interactive practice exercises and weekly skill practice goals, and a quiz to evaluate the understanding of the module content. The IMPACT dashboard landing page displays personalized information for each participant, including a badge for each completed module, stars for completion of practice exercises, weekly averages of daily steps and step goals in graphical form, and a link to weekly coach messages.

Participants receive links to daily digital surveys through MMS text messages on their mobile phone at a participant-selected time for the duration of treatment. Surveys assess pain intensity, pain interference, pedometer-measured step counts, sleep quality, sleep duration, craving to use opioids, self-efficacy, MOUD adherence, and questions regarding the practice of the treatment skills. Based on our previous work, digital surveys are estimated to take no longer than 2 minutes to complete. Coaches review summaries of the daily survey data through a web portal that displays survey responses from the past week (as weekly averages or individual days). The coach records an audio message based on the participant-provided responses to the current week’s surveys, as well as previous weeks, that is uploaded to the participant’s IMPACT dashboard. The coach’s message provides reinforcement of skill practice and goal attainment and highlights associations between participants’ daily responses and trends in weekly averages over the course of treatment. Coaches receive training in message preparation and attend weekly supervision with a clinical psychologist. Participants are not able to directly respond to the coach’s feedback through the program but are able to indirectly communicate with the coach by leaving a message with the research assistant during their weekly clinic visit, who can relay the message to the coaches.

#### TAU (Control)

Participants in both conditions receive TAU for the buprenorphine and methadone programs at participating MOUD clinics, which includes regular medication management by the clinic physician and regular individual and group counseling sessions delivered on-site by counselors employed at participating MOUD clinics. Participants randomized to IMPACT+TAU and TAU will receive MOUD dosing as indicated by their OTP clinician in conjunction with regular meetings as dictated by their treatment plan. Both groups are free to access any OTP clinic resources, including prescriptive services for mental health issues, group treatment, and individual counseling as offered at each recruitment site.

### Assessment Visits

The study includes a baseline visit followed by brief weekly visits during treatment (up to 12 weeks) and a 3-, 6-, and 9-month postrandomization assessment visit. Neither participants nor research assistants are blinded to group assignments. However, outcome measures are obtained through self-report using REDCap digital assessments, and MOUD retention will be verified with MOUD clinic records, limiting the potential for bias. Participants complete the baseline, weekly visit, posttreatment, and follow-up measures at their outpatient MOUD clinic to facilitate the collection of a urine sample for urine toxicology analysis (Table 1). After baseline, if in-person attendance is not possible, study measures may be completed remotely. Remote participation is facilitated by the use of digital data collection, videoconferencing, or the telephone.

**Table 1:** Study measures.

Measure			Items		Baseline		Weekly		3 months		6 and 9 months
**Baseline measures**											
	Demographic information, MOUD^a^ information, and clinical characteristics of chronic pain			✓							
	MINI^b^ International Neuropsychological Interview [59]	Interview		✓							
	Graded Chronic Pain Scale–Revised (frequency, severity, and impact of pain) [53]	6		✓							
	Addiction Severity Index [60]	Interview		✓				✓		✓	
**Primary outcome (in 3 months)**											
	Buprenorphine retention	none		✓				✓		✓	
**Secondary outcome measures**											
	PROMIS^c^ Pain Interference 6a [61]	6		✓				✓		✓	
	MOUD Adherence^d^	None		✓		✓		✓		✓	
	The Craving Scale [62]	3		✓				✓		✓	
	Timeline Follow-Back Substance Use Calendar [63]	Interview		✓		✓		✓		✓	
	Urine toxicology	None		✓		✓		✓		✓	
	NRS^e^ Pain Intensity Rating [64]	1		✓				✓		✓	
	PROMIS Physical Function 6b [65]	7		✓				✓		✓	
	Pain Catastrophizing Scale [66]	14		✓				✓		✓	
	Pain Self-Efficacy Questionnaire [67]	11		✓				✓		✓	
	PROMIS Sleep Disturbance 6a and Duration [68]	9		✓				✓		✓	
**Other measures**											
	Brief Symptom Inventory [69]	18		✓				✓		✓	
	Cold pressor task	None		✓				✓		✓	
	PHQ-4^f^ (brief screen for anxiety and depression) [70]	4		✓				✓		✓	
	Program and Client Cost-Substance Abuse Treatment (treatment use) [71]	Interview		✓				✓		✓	
	IMPACT^g^ engagement data (IMPACT+TAU^h^ only)	None				✓					

^a^MOUD: medications for opioid use disorder.

^b^MINI: Mini-International Neuropsychiatric Interview.

^c^PROMIS: Patient Reported Outcomes Measurement Information System.

^d^Adherence will be assessed with the Timeline Followback for daily MOUD use for the previous week, clinic dispensing records, and urine toxicology screen for buprenorphine or methadone collected at each weekly assessment visit.

^e^NRS: Numerical Rating Scale.

^f^PHQ-4: Patient Health Questionnaire-4.

^g^IMPACT: Integrating the Management of Pain and Addiction via Collaborative Treatment.

^h^TAU: treatment as usual.

### Primary Outcome Measure (Retention in MOUD)

Retention is defined as dichotomous enrollment in MOUD (ie, yes or no), with evidence of MOUD uptake in the week before the 3- (primary), 6-, and 9-month time points. Using data from the 7 days before the 3-month time point, dichotomous enrollment in MOUD services will be verified by clinic records and evidence of MOUD compliance will be assessed using clinic records and self-report. When these sources disagree, clinic records will be used to determine compliance. Retention will be a binary outcome, with 1=retained (conditions met) and 0=not retained.

### Secondary Treatment Outcome Measures

We will also collect OUD (ie, MOUD adherence, craving, and substance use) and pain (ie, pain interference, physical functioning, pain intensity, sleep disturbance, pain catastrophizing, and pain self-efficacy) secondary outcome measures. These outcomes evaluate other important treatment effects that have an evidence base in OUD and chronic pain treatment studies and will provide a more detailed assessment of possible treatment outcomes. Outcome measures are detailed in Table 1.

### Other Measures

To better characterize other treatment effects, we will also evaluate general psychological symptoms, depression symptoms, anxiety symptoms, OTP treatment use and cost, cold pressor task (pain threshold), and digital treatment engagement (only participants randomized to IMPACT+TAU).

### Data Sharing

Data collected during baseline, post treatment, and follow-up visits will be made available in compliance with the HEAL Public Access and Data Sharing policy. The study is registered in ClinicalTrials.gov and is in the process of being registered in the HEAL Data Platform. Once data collection is complete, 1 master data file will be created that includes all variables necessary to address the primary study aims and hypotheses. All analyses will be performed using the master data file. The final master data set will then be submitted to the appropriate HEAL-approved repository. All data will be deidentified before submission using procedures that are in compliance with the HIPAA (Health Insurance Portability and Accountability Act), the Yale Human Investigation Committee, and NIH standards.

### Qualitative Interviews

Among those randomized to IMPACT+TAU, individual interviews (N=34) will be conducted at 3-month follow-up with a sample of completers (n=17) and noncompleters (n=17). This sample size should be sufficient to achieve saturation on interview themes; however, if saturation is not reached, additional participants will be interviewed until saturation is attained [72]. Completers will be defined as any participant that engages in 7-9 IMPACT modules. Noncompleters will be defined as any participant who completed between 1 and 4 IMPACT modules. Interviews will take place on the internet or in person, be digitally recorded, and be transcribed. All respondents will be asked a series of open-ended questions to probe participant experiences with IMPACT, including barriers or facilitators to engagement and how IMPACT influenced their ability to manage pain and OUD. Semistructured questions will elicit feedback on various features of the intervention, including the various module topics (Figure 3), the walking component, impressions of the weekly personalized feedback, the IMPACT platform, study outcomes, daily surveys, and combined emphasis on pain or OUD.

### Data Analyses

#### Quantitative Data Analysis

Data will be reported for each treatment arm and overall at all time points in the study. Summary descriptions of recruitment rates, attrition, daily survey completion, and treatment outcomes will be calculated. The baseline demographic characteristics of both groups will be summarized.

We will perform a true intent-to-treat analysis of the primary MOUD retention outcome that will include all randomized participants and use logistic regression models to assess differences in MOUD retention by treatment condition at the 3-month time point. MOUD type (ie, buprenorphine or methadone) will be specified as a covariate. Similarly, the logistic regression model will be used to assess differences in MOUD retention by treatment condition at each follow-up time point (ie, 6 and 9 months post randomization).

In addition to retention, we will run secondary analyses to evaluate differences in several OUD (ie, MOUD adherence, craving, and substance use) and pain (ie, pain interference, physical functioning, pain intensity, sleep disturbance, pain catastrophizing, and pain self-efficacy) measures by treatment condition and by time using multilevel longitudinal models with MOUD type (ie, buprenorphine or methadone) specified as a between-person covariate.

#### Qualitative Data Analysis

Analysis of qualitative data will be conducted using Atlas.ti, a qualitative software program allowing fluid interaction of data across types or sources [73]. A qualitative-descriptive approach is suitable when information is needed to develop, summarize, or refine an intervention [74]. A constant comparative approach with sequential analysis will be used to reach a thematic consensus. Memos, field notes, and debriefing notes after each interview will also be incorporated into the analysis [75]. Each transcript will first be read in its entirety and then coded, proceeding from line-by-line in vivo codes to more broad codes and themes, comparing data across participants [75]. Coding will be completed independently by 2 researchers and compared, and final themes will be arrived at through consensus. Findings will be summarized and presented in tables. It is expected that the collected data will reflect mechanisms underlying engagement and participant perspectives of IMPACT.

## Results

The funding for the clinical trial component of the study (phase 2) was obtained in June 2022, and the trial study protocol that included changes from phase 1 was approved by the institutional review board in January 2022. Enrollment began on July 15, 2022, at a single outpatient clinic; a second outpatient clinic was added on March 6, 2023; and a third site is expected in January 2024. Enrollment is expected to continue until June 2024. Final data are expected to be collected in March to May 2025, and the primary results of the study are expected to be published in March to May 2026.

## Discussion

### Summary of Study Significance

Given the prevalence of OUD and the increased mortality by overdose, the provision of MOUD and support for retention in MOUD are critical. Only a third of individuals receiving MOUD are retained in treatment after 6 months. Clearly, many people receiving MOUD require additional support to remain in treatment and attain its full benefit. Chronic pain, a common co-occurring condition among those receiving MOUD, interferes with MOUD retention and diminishes functioning and quality of life. An asynchronous digital integrated treatment for addiction and chronic pain, such as IMPACT, holds promise to increase access to evidence-based behavioral treatment in MOUD clinical care. IMPACT is designed to be accessible, low-burden, and easily integrated into MOUD clinics despite their limited resources.

### Benefits of Daily Surveys

Importantly, IMPACT collects individual responses through daily surveys that are used to generate weekly personalized feedback for participants. These data are the foundation of personalized coach feedback that may enhance engagement with treatment, which has been suggested in previous reviews [76] and yield relatively fine-grained data on patient reports of pain and pain interference, craving to use opioids, sleep problems, and physical activity for a comparatively large sample of individuals enrolled in MOUD. Daily ratings of pain and pain fluctuations in this population are unique, and the data generated will be critical in addressing key gaps in the literature, including understanding fluctuations in relationships between pain and pain interference, drug craving, treatment retention, sleep, and functioning in this population. These data may provide actionable information for further treatment refinement.

### Benefit of Digital Interventions in the MOUD Setting

An integrated, digital intervention offers 2 particularly attractive benefits in the MOUD setting: a means to both (1) obtain treatment in a less stigmatizing manner and (2) provide treatment with high fidelity to evidence-based protocols without adding staff. Individuals receiving MOUD report high levels of stigma that can significantly impact the course of treatment [77-79]. Increased stigma is a primary barrier to engaging in MOUD treatment and is present even among addiction providers. In a recent qualitative study of MOUD providers, the perception of psychosocial interventions is mixed, with some providers suggesting that MOUD undermines the effectiveness of behavioral treatment while others argue that any provision of behavioral treatment is not a necessary component of MOUD treatment [80]. Furthermore, integrating evidence-based psychotherapies, such as CBT, into MOUD clinical care has been met with mixed results [81-83]. Some researchers have posited that the inconsistent findings are likely related to methodological flaws in previous research, including low fidelity and an inadequate control group [42]. IMPACT treatment materials can be delivered with a high degree of fidelity and consistency, effectively removing a lack of fidelity as a potential driver of outcomes in this study. The patient demands of MOUD clinical care often preclude trained providers from delivering behavioral treatment [84]. Technology-based and digital interventions, such as IMPACT, can address these barriers by offering treatment with high fidelity that can be completed outside the clinic, further reducing the possible stigma of discussing problems related to addiction and pain in MOUD settings.

### Limitations

There are several limitations to the current protocol. First, as is common in behavioral intervention trials, participants will be unblinded to the condition once randomized. However, all participants and researchers will be blind to the condition when completing baseline structured interview measures. The primary outcome is based on objective measures and clinic attendance and, therefore, is less vulnerable to biases associated with being unblinded. Other treatment outcomes are collected through self-reported responses in REDCap, which also limits the potential for bias. There is also no attention control for IMPACT. We chose to use TAU as the control condition to maximize our ability to determine the efficacy of adding a digital treatment to the existing gold standard treatment (ie, MOUD). Next, participants randomized to IMPACT may not complete all daily surveys, resulting in missing data. While we expect that participants will miss surveys, completing the surveys benefits participants because they provide personalized feedback; and even 1-2 surveys per week can provide insight for weekly coach feedback. Additionally, previous research on COPES has shown high survey completion [85], which is consistent with findings from other asynchronous chronic pain interventions using daily surveys [86]. Finally, participants in both groups may receive other pain and addiction treatments both within and outside their MOUD clinic. We will inquire about additional treatment at weekly visits during treatment to account for any effects that could be attributed to other interventions.

### Conclusions

Access to behavioral treatment for individuals with OUD and chronic pain is a critical gap in MOUD care. Offering access to a combined pain and addiction treatment may help retain individuals in MOUD while improving clinical outcomes for people who otherwise may not receive treatment. Digital treatments are especially well-suited to MOUD treatment settings because they reduce stigma and provide access to high fidelity, evidence-based behavioral treatment.

## Appendix

Multimedia Appendix 1 Peer-viewer report from the National Centre for National Center for Complementary and Integrative Health (NCCIH).

## Abbreviations

BRIM: Behavioral Research to Improve Medication-Based Treatment
CBT: cognitive behavioral therapy
CBT4CBT: Computer-Based Treatment for Cognitive Behavioral Therapy
COPES: Cooperative Pain Education and Self-Management
DSM-5: Diagnostic and Statistical Manual of Mental Disorders (Fifth Edition)
HEAL: Helping to End Addiction Long-Term
HIPAA: Health Insurance Portability and Accountability Act
IMPACT: Integrating the Management of Pain and Addiction via Collaborative Treatment
MOUD: medications for opioid use disorder
NIH: National Institutes of Health
OTP: opioid treatment program
OUD: opioid use disorder
REDCap: Research Electronic Data Capture
TAU: treatment as usual

## Data Availability

Deidentified data sets generated during the course of this study will be made available on the Helping to End Addiction Long-Term (HEAL) Data Platform through a HEAL-approved repository.
