# Supplementary material for: Efficacy of Integrating the Management of Pain and Addiction via Collaborative Treatment (IMPACT) in Individuals With Chronic Pain and Opioid Use Disorder: Protocol for a Randomized Clinical Trial of a Digital Cognitive Behavioral Treatment
Source: JMIR Res Protoc. 2024 Mar 20;13:e54342. doi: 10.2196/54342 (PMC10993119; doi:10.2196/54342)
Supplement: Multimedia Appendix 1 [file resprot_v13i1e54342_app1.pdf]

SUMMARY STATEMENT
(Privileged Communication)

PROGRAM CONTACT:
Dr. David Clark
301-827-1916
dave.clark@nih.gov

Release Date: 07/02/2019
Revised Date:

Application Number: 1 R61 AT010619-01

Principal Investigators (Listed Alphabetically):

CARROLL, KATHLEEN M. (Contact)
HEAPY, ALICIA

Applicant Organization: YALE UNIVERSITY

Review Group: ZAT1 SM (48)
National Center for Complementary and Integrative Health Special Emphasis Panel
HEAL Initiative: Behavioral and Social Interventions to Improve Adherence to Medication Assisted Treatment for Opioid Use Disorders

Meeting Date: 06/21/2019
Council: AUG 2019
Requested Start: 09/01/2019

RFA/PA: AT19-006
PCC: CLARKD
Dual PCC: CM/SQD
Dual IC(s): DA

Project Title: Adapting Web-based CBT to improve adherence and outcome for individuals with opioid use disorder and chronic pain treated with opioid agonists
SRG Action: Impact Score:29
Next Steps: Visit https://grants.nih.gov/grants/next_steps.htm
Human Subjects: 30-Human subjects involved - Certified, no SRG concerns
Animal Subjects: 10-No live vertebrate animals involved for competing appl.
Gender: 1A-Both genders, scientifically acceptable
Minority: 1A-Minorities and non-minorities, scientifically acceptable
Age: 3A-No children included, scientifically acceptable

Project Year	Direct Costs Requested	Estimated Total Cost
1	299,994	490,449
2	299,964	490,400
3	592,778	969,111
4	589,647	963,992
5	584,968	956,343
TOTAL	2,367,351	3,870,295

1R61AT010619-01 CARROLL, KATHLEEN
RESUME AND SUMMARY OF DISCUSSION: This R61 application titled “Adapting Web-based CBT to improve adherence and outcome for individuals with opioid use disorder and chronic pain treated with opioid agonists” is submitted in response to RFA-AT-19-006 “HEAL Initiative: Behavioral Research to Improve MAT: Behavioral and Social Interventions to Improve Adherence to Medication Assisted Treatment for Opioid Use Disorders (R61/R33)” by Yale University with Dr. Kathleen Carroll and Dr. Alicia Heapy as principal investigators. The application aims to establish and refine a new web-based intervention that targets pain management in patients with OUD. This innovative and significant application addresses access to behavioral therapy as a key barrier to treatment and if effective, it could easily be integrated into clinical care. Chronic pain is a significant risk factor for OUD, and patients with chronic pain and OUD usually have poorer MAT adherence. Further, cognitive behavioral treatment (CBT) for chronic pain (CP) has been recommended as a first-line therapeutic approach to reduce pain and improve function. A web-based intervention targeting chronic pain and OUD is novel, as is the use of interactive voice response (IVR) as an investigative tool. Analysis of treatment effects on pain fluctuations is also novel. However, a barrier to implementing this intervention could be the lack of access to internet or mobile phones; the focus solely on the population with comorbid chronic pain and OUD reduces generalizability. This is an exceptionally strong research team with complementary areas of expertise in areas highly relevant to this study such as developing psychological interventions and disseminating them using computer-based platforms. A very strong environment with a large population pool and mostly advanced facilities are available, albeit this may not necessarily be representative for other similar treatment programs across the country. Overall, this study has a strong analytical approach and well-designed primary and secondary outcomes. The use of pain interference as a measure is clinically relevant and likely to emphasize group differences. Adequate randomization methods were proposed. The R61 phase uses a previously validated model of treatment for a similar web-based intervention, increasing the success probability. A logical sequence of studies is proposed with a thorough 12 months plan outlined. The small feasibility study described could enhance the intervention even further. The R33 phase solid methodological design will increase rigor and transparency with a well-designed plan to monitor and manage clinical deterioration. Clear inclusion and exclusion criteria, well validated outcome measures, and proper blinding strategies were proposed. The recruitment target will be easily reached. A few minor to moderate generally fixable weaknesses were noted. The small sample size for the feasibility study might increase the possibility for bias, which could also be increased by the heavy interaction of study subjects with the research team. The high burden on participants could increase attrition; this could also inadvertently select the more motivated population segment to remain in the study, thus risking a lower generalizability of the study. The chronic pain diagnosis and concurrent medications were not very carefully considered in the data analysis plans. An appropriate plan for how to address the likely high amount of missing data was not included. Some concerns were noted in the statistical data analysis that were viewed as readily addressable. The R61 to R33 transition milestones were described but were generally judged as somewhat underdeveloped. In conclusion, the numerous strengths of this application that could have a high impact on the field, coming from a very strong research team and with a rigorous design particularly for the R61 phase outweighed the predominantly minor weaknesses stemming mainly from fixable issues with the study design, potential generalizability barriers as well as barriers inherent to technology access and resulted in generally high enthusiasm for this application.

DESCRIPTION (provided by applicant): Chronic pain is common among individuals treated with opioid agonist therapies (OAT; buprenorphine or methadone) and is associated with poorer opioid use disorder (OUD) outcomes, including higher attrition and higher levels of drug use. Treatment of chronic pain in individuals who are maintained on OAT is complex, as presence of chronic pain is associated with greater frequency of psychiatric disorders as well as sleep problems and other comorbidities that can contribute to poorer outcomes. Given the large number of individuals with chronic pain seeking OAT, standardized, lower-cost, and easily disseminable approaches to treat individuals in OAT who

have chronic pain are needed and, if demonstrated to be effective in this population, would have high public health significance. In response to RFA-AT-19-006, we plan to develop and pilot test an integrated, web-based cognitive behavioral approach (R61 phase), and then conduct a randomized clinical trial evaluating its efficacy relative to standard care in a large and diverse sample of individuals with chronic pain treated with buprenorphine or methadone (R33 phase). The new program will retain key components of Dr. Carroll's computer-based training for cognitive-behavioral therapy (CBT4CBT), including its emphasis on teaching cognitive and behavioral coping skills in an engaging way and focus on the 5 A's of MAT (Adherence, Attendance, Abstinence, Alternate Activities and Accessing support); it will add components from Dr. Heapy's COPES (Cooperative Pain Education and Self-Management) intervention (self-management of chronic pain, with IVR monitoring of pain intensity and interference, physical activity, and skills practice) and modify existing CBT4CBT modules to address the complex interplay between pain and drug use in this population, emphasizing the development of generalizable skills. In the R33 phase, we will conduct a randomized clinical trial evaluating CBT4CBT-COPES in a diverse sample 160 of individuals enrolled in agonist treatment (methadone or buprenorphine) who have chronic pain, in a 3-month randomized clinical trial with a 6-month follow-up, comparing it to standard treatment alone. The primary retention outcome will be adherence with agonist treatment; the primary pain outcome will be the PROMIS 6-item Pain Interference Short Form.

PUBLIC HEALTH RELEVANCE: Chronic pain is common among individuals treated with opioid agonist therapies (OAT; buprenorphine or methadone) and is associated with poorer opioid use disorder (OUD) outcomes, including higher attrition and higher levels of other drug use. The goal of this project is to develop and pilot test an integrated, web-based cognitive behavioral approach (R61 phase), and then conduct a randomized clinical trial evaluating its efficacy relative to standard care in a large and diverse sample of individuals with chronic pain treated with buprenorphine or methadone (R33 phase).

CRITIQUE 1

Significance: 2
Investigator(s): 1
Innovation: 2
Approach: 3
Environment: 3

Overall Impact:
This application seeks funding to support a program of research to develop, refine, and test a novel web-based intervention that addresses pain management in patients with OUD. The topic being addressed is quite important clinically. The results of the study could impact current clinical practice since the intervention is potentially disseminable. The investigative team is outstanding and has been highly productive. The academic environment is strong. The application is well-written and the studies proposed have many positive features.
R61 Phase: The sequence of studies will enable the investigators to further develop and refine a novel intervention. The sequence is logical with one activity building on the prior one. Well described core principles will guide intervention development and multiple stakeholders will provide input and feedback. The milestones provided are somewhat vague and not well defined operationally. This is considered a minor concern since the level of detail provided in the description of the activities could enable the investigators to develop clearer milestones. The feasibility trial proposed is rather small to inform the larger clinical trial.
R33 Phase: The randomized clinical trial is well designed with many positive methodological features. A strong plan for monitoring and handling clinical deterioration is included. Some concerns are noted. These include the failure to include a control condition that adequately controls for attention and level of

interaction with the project, high participant burden which could heighten attrition and reduce the quality of data gathered, the possibility that study staff at the clinic sites could interact with intervention arm participants in ways that could confound interpretation of the findings, and lack of appropriate attention to patient diagnosis and other ongoing medications. Finally, it is not clear that conducting the proposed study at the clinical facilities described is the best option, since the investigators' prior work in those facilities might have impacted usual practice in ways that might make it harder to detect a treatment effect.

Summary: Overall this is a well written application that tests a novel web-based intervention. Its results could have an impact on clinical practice in this field. Although some concerns are noted, it is felt that these could be satisfactorily addressed and that useful findings could thereby be achieved.

1. Significance:
Strengths
- This project addresses an extremely important and timely clinical problem: the management of patients with chronic pain and opioid use disorder.
- The study proposed is important because it addresses a key barrier to effective treatment of this population: access to high quality and empirically validated behavioral approaches to treating pain and opioid use disorder.
- The results of this study could have an important impact on clinical practice in that, if effective, the intervention being tested could be relatively easily integrated into clinical care. It thus could not only increase access to treatment but also improve outcomes for patients with chronic pain involved in programs for opioid use disorder.
- The proposed clinical trial would provide new data on the efficacy and safety of a novel web-based intervention. The data that will be provided are essential to collect before such an intervention could be considered part of clinical care.
- The proposed project builds upon a strong foundation of preliminary data.
- Because the inclusion criteria are broad, the likelihood that findings will be generalized to other populations enrolled in other opioid use disorder treatment programs is high.
- The two primary outcomes are both very important ones: adherence and pain. It is well known that these outcomes are related to long-term outcomes of treatment for patients with chronic pain and opioid use disorder, however little is known about how a web-based behavioral intervention will impact these outcomes. Thus, the trial could fill an evidence gap.

Weaknesses
- The study sites involved are very high-quality ones that have participated in prior academic research. It is unclear whether the treatment approaches/programs being used are comparable to those offered at other programs that may offer much less state of the art treatment. This raises concerns about the generalizability of findings across treatment programs.

2. Investigator(s):
Strengths
- This is an exceptionally strong research team. It features one principal investigator with extensive experience with behavioral treatments for individuals with drug-related problems and another principal investigator with a track record of testing behavioral treatments for chronic pain and publishing the results of their studies. In prior studies they both have been successful in both recruiting and retaining study participants. Both principal investigators have directed high quality randomized clinical trials in the past. Both have shown a high level of productivity. Most key members of the team are experienced and have worked with one or more of the principal investigators. As a group, they have complementary areas of expertise (e.g. computer-based delivery of behavioral treatments (Dr. Martino), psychopharmacological treatments for patients with opioid use disorder (Dr. Sofuoglu), addiction medicine (Dr. Becker), and clinical expertise in the treatment of patients with substance abuse (Dr. Hamilton). The research team has

successfully collaborated in the past with the clinical sites (treatment clinics at the Liberation Program).

Weaknesses

- No weaknesses were noted.

3. Innovation:

Strengths

- The investigators make a strong case for the innovation of this project.
- A web-based intervention based on empirically validated treatment components and that targets both chronic pain and opioid use disorder is novel.
- The plan to use IVR gathered data is also novel.
- An analysis of effects of treatment on pain fluctuations is novel.
- The project's potential impact on public health is innovative.

Weaknesses

- CBT approaches to be included in the web-based program are not novel in that they are similar in content to those used by these and other investigators in the past.
- The theoretical significance of the application is rather under-developed.
- The RCT study design is straightforward and not that novel.
- The outcome measures, likewise, are fairly standard for the field.

4. Approach:

R61 Phase Strengths

- The R61 phase is conceptualized as a Stage 1 project using an established model of treatment development (the Stage Model).
- The activities outlined are appropriate and similar to those successfully used by this group in their past development of web-based interventions. This increases the feasibility of this phase of the study.
- The panel of experts that will participate in an initial meeting regarding intervention development is highly skilled and has expertise in relevant domains.
- The two additional panels to be convened to develop vignettes and provide suggestions for the new program involve very appropriate personnel: i.e. individuals with OUD and chronic pain and clinicians for the Liberation program.
- The investigators already have identified a key set of modules that will be the focus of intervention development efforts in the R61 phase (a new introductory module that provides a rationale for addressing both chronic pain and opioid use disorder, two new modules (pain and sleep, and CBT techniques for pain and opioid use problems), and modification of seven existing modules.
- A 12 months plan for developing the integrated intervention is outlined. A set of strong core principles is outlined to guide this work and multiple examples of relevant content for the intervention are outlined.
- A very small feasibility trial is also described (N=5) which focuses on relevant information that could be used to shape the intervention.

R61 Phase Weaknesses

- Many of the milestones listed are vague and not well detailed ("Milestone: minutes from meetings; refined plans for adaptation". The lack of a more comprehensive set of well-articulated and operationally-defined milestones is a concern. Without such clear milestones it will be difficult to accurately track progress during the R61 phase.
- The sample size for the feasibility trial seems quite small for a project of this magnitude. When gathering such data from only 5 individuals the likelihood of bias is increased.

R33 Phase Strengths

- The study design has numerous methodological strengths that increase rigor and transparency. These include random assignment to treatment conditions, a clear set of inclusion and exclusion criteria, a generally well validated set of self-report and other outcome measures, assessment of outcomes by individuals who are blind with respect to treatment condition, collection of data at both pre and post-treatment and at follow up, and use of a number of best practices to enhance retention.
- The plan for monitoring and handling clinical deterioration is well developed.

R33 Phase Weaknesses

- Participants in the treatment intervention will receive a great deal of attention and interaction with the research project. There is no actual control condition to control for the effects of attention.
- Participant burden is quite high both in terms of assessment and treatment and may lead to higher attrition rates than anticipated. Conversely, participants who are retained in the study despite the significant burden may be those who are highly motivated and perhaps unrepresentative of the larger population to which the researchers aim to generalize their findings.
- Participants in the intervention arm of the study can work on the intervention program at the clinic sites. In the course of doing so they may well reach out to and involve clinic staff who are likely to be willing/want to assist them with the program. The support and assistance of clinicians (many of who are likely to be trained in CBT) is not being tracked but could confound interpretation of study findings.
- Treatment contamination may occur as patients in the intervention arm begin to talk about their online experiences and learning.
- There is a lack of attention to patient chronic pain diagnoses thatcould impact study findings.
- Little is said about how use of other medications will be monitored and considered in the data analyses.

5. Environment:
Strengths

- The investigators appear to have access to a large potential population for the proposed program of research.
- They have a strong track record of enrolling and retaining participants from these and other centers in the past.
- The Liberation Program sites appear to be supportive of clinical trials research thereby enhancing the feasibility of this project.
- The researchers' academic settings appear to be quite supportive of research.

Weaknesses

- The Liberation Program sites appear to offer state of the art treatment. These sites may however not be that representative of the larger population of treatment programs across the country.
- The Liberation Program services appear to be very sophisticated. In addition, it is noted that the Liberation Program has collaborated with this group of researchers in the past. Thus, it is highly likely that cognitive behavioral interventions already are in place and will be available at the study sites. Along these lines, it is noted that study participants will have ready access to regularly available services at these sites. The ready availability of face to face CBT (which in this population likely focuses on both pain and opioid use issues) may make interpretation of the findings difficult.

Study Timeline:
Strengths

- The timeline for the R33 Phase appears to be reasonable.

Weaknesses

- The timeline for the R61 Phase appears to be optimistic. It may be difficult to complete all of the tasks outlined in the time available.

R33 to R61 Transition Milestones:
Strengths
- Some milestones are provided for the R61 phase.

Weaknesses
- As noted earlier the R61 milestones are not well developed or articulated.
- Challenges to the R61 milestones and strategies for overcoming these challenges are not well developed.

Protections for Human Subjects:
Acceptable Risks and/or Adequate Protections
- The investigators appear to be aware of the risks involved in conducting this type of research and have outlined steps to minimize these.

Data and Safety Monitoring Plan (Applicable for Clinical Trials Only):
- Acceptable
    - The DSMP is well developed and reasonable for a study of this type.

Inclusion Plans:
- Sex/Gender: Distribution justified scientifically.
- Race/Ethnicity: Distribution justified scientifically.
- For NIH-Defined Phase III trials, Plans for valid design and analysis: Not applicable
- Inclusion/Exclusion Based on Age: Distribution justified scientifically.

Vertebrate Animals:
Not Applicable (No Vertebrate Animals)

Biohazards:
Not Applicable (No Biohazards)

Resubmission:
Not Applicable

Renewal:
Not Applicable

Revision:
Not Applicable

Applications from Foreign Organizations Not Applicable.:
Not Applicable (No Foreign Organizations)

Select Agents:
Not Applicable (No Select Agents)

Resource Sharing Plans:
Acceptable

Authentication of Key Biological and/or Chemical Resources:
Not Applicable (No Relevant Resources)

Budget and Period of Support:
Recommend as Requested

- The budget requested appears reasonable given the scope of work outlined.

CRITIQUE 2

Significance: 2
Investigator(s): 2
Innovation: 2
Approach: 3
Environment: 1

Overall Impact:
This project by an excellent team of investigators is to (1) develop and pilot test an integrated, web-based cognitive behavioral approach (R61 phase) to be used in opioid agonist treatment to improve retention and chronic pain; and then, (2) to conduct a randomized clinical trial evaluating its efficacy relative to standard care in a large and diverse sample of individuals with chronic pain treated with buprenorphine or methadone (R33 phase). There are numerous strengths to this application including the investigative team, the excellent intellectual environment, a strong track record with CBT development, a strong theoretical basis to the intervention, and a rigorous design in the R61 phase. The enthusiasm is only slightly diminished by the lack of a current office-based buprenorphine clinic site for the R61 phase that may limit generalizability and significance, possibly diminished feasibility due to lack of reliable phone service in this population, and lack of clarity on when patients may enroll (before or after 30 days on OUD treatment).

1. Significance:
Strengths

- There is a significant possibility that this intervention will provide increased availability of CBT for this challenging population with poorer OUD treatment outcomes.
- The high occurrence of other conditions in persons on agonist treatment for OUD that may benefit from CBT even further.
- The lack of trained clinicians in CBT is a problem that is unlikely to be fixed any time soon.

Weaknesses

- Access to the Internet is problematic in many areas of the country, especially rural areas. This may negate some of the significance of a successful project.

2. Investigator(s):
Strengths

- This is a great and productive research team.
- The strong track record of team work amongst the multiple principal investigators was noted.
- A great infrastructure is available to produce and perfect the intervention.

Weaknesses

- There was no identified post-doctoral fellow in the application.
- The fact that Dr. Carroll is owning the copyright of CBT4CBT, the intervention that will provide the basis for the web-based intervention to be developed here is of some concern. This concern is however somewhat mitigated by the fact that the application has been submitted by Yale University.
- The effort on the investigators at the VA site was not clearly stated and made it hard to determine if they had enough time to commit the project.

3. Innovation:

Strengths
- Although the use of smart phones has decreased the interest in web-based platforms, the need for a technology-based intervention is needed for the population of patients with OUD and chronic pain – who are often isolated.
- This reviewer appreciates the need to keep the CBT4CBT intervention engaging.
- Although ambitious, it is useful to develop a CBT protocol aiming to improve affective symptoms, sleep quality, and OUD treatment outcomes.

Weaknesses
- The need for ongoing access to a telephone is problematic in this population. Although most patients have a phone, the number is often changing and the lack of minutes is a constant threat towards the end of each month.

4. Approach:
Strengths
- The strong track record of the investigators gives great evidence that the project would be successful in producing an effective intervention and a controlled trial providing high quality outcome data on its efficacy.
- Dr. Jamison is an excellent choice for a pain-related consultant.
- The use of pain interference is clinically relevant and more likely to show a difference between conditions compared to pain ratings.
- The inclusion of sleep in the intervention is very important.

Weaknesses
- The choice of primary outcomes (continuous enrollment in agonist treatment at 3 months) is rather conservative.
- The wide variety of pain syndromes allowed may reduce the trial efficacy as chronic headaches and chronic pain from opioid use (hyperalgesia) are not known to be improved by CBT.
- There is a moderate concern about feasibility due to lack of evidence that patients have continuous access to phone throughout the month for the interactive voice response (IVR) part of the intervention. The COPE trial excluded persons with SUD.
- It is unclear why persons on dialysis are excluded as this group has high prevalence of chronic pain and opioid use.
- The use of 4 matching variables may be challenging to implement with a relatively small sample size as proposed for the RCT.
- Lack of an adequate plan for missing data, which is likely to be high.
- The preliminary data for the COPES trial was -1 in mean pain ratings and similar reductions in pain interference. The effect of each intervention is most likely going to be small to moderate. Whether this size is effective in improving MAT treatment retention and outcomes is an important question to be answered though.

5. Environment:
Strengths
- Strong evidence and adequate budget to produce the web-based programming.
- The access to OnCore is a strength.
- The proposed sites appear to have an adequate patient population from which to recruit in the given time frame.

Weaknesses
- No major weaknesses noted.

Study Timeline:
Strengths
- There are clearly stated recruitment efforts for R33 phase.

Weaknesses

- The timeline is not well developed and lacks objective milestones.

R33 to R61 Transition Milestones:
Strengths
- The development of the intervention will be self-evident.
- The number of participants (N=5) is acceptable for pilot work.

Weaknesses
- Transition milestones are underdeveloped. Minutes from a meeting as part of the refined plans for adaptation could be helpful (or not).
- The range in ratings of usability was not clear What does a 3.5 mean?

Protections for Human Subjects:
Acceptable Risks and/or Adequate Protections
- Data and Safety Monitoring Plan (Applicable for Clinical Trials Only):Acceptable

Inclusion Plans:
- Sex/Gender: Distribution justified scientifically.
- Race/Ethnicity: Distribution justified scientifically.
- For NIH-Defined Phase III trials, Plans for valid design and analysis: Not applicable.
- Inclusion/Exclusion Based on Age: Distribution justified scientifically.

Vertebrate Animals:
Not Applicable (No Vertebrate Animals)

Biohazards:
Not Applicable (No Biohazards)

Resource Sharing Plans
Acceptable
- Should ensure that the product would be available at cost if it remains under copyright.

Authentication of Key Biological and/or Chemical Resources:
Not Applicable (No Relevant Resources)

Budget and Period of Support:
Recommended budget modifications or possible overlap identified:
- The Singlebrook Technologies Inc web development firm was not mentioned in the budget justification. Therefore, will the budget be adequate?
- Unclear if the investigators have budgeted for or access to cold pressor testing equipment.

CRITIQUE 3

Significance: 2
Investigator(s): 1
Innovation: 2
Approach: 2
Environment: 1

Overall Impact:

R61 Phase: The R61 Phase will begin with an expert panel who will review CBT4CBT-COPES integration followed by stakeholder panels for patients and providers. These panels will guide development of CBT4CBT-COPES and existing CBT4CBT modules will be used as a foundation for the integrated treatment. This will then be tested in a small (n=5) pilot study.**R33 Phase**: The R33 Phase will include a RCT comparing CBT4CBT-COPES to treatment as usual in 160 participants over 3 months (followed by a 6-month follow-up). Primary outcomes include OAT retention and the PROMIS Pain Interference Short Form.**Summary**: The proposed study comes from a very strong and experienced research group and is based on the CBT4CBT platform (a tested and efficacious mechanism for pain management using an accessible online format) combined with COPES. The primary weakness of this work is that it assumes a need to combine these treatments without testing the treatments on their own in a Pain-OUD sample. This work may not be necessary. However, the strengths of the R61 Phase (stakeholder interview and expert panel review) mitigate this concern significantly because if the current content is adequate, these mechanisms should be able to identify that. Another small weakness is the decision to combine COPES and CBT4CBT given the already demonstrated benefit of CBT4CBT-Bup.

1. Significance:
Strengths
- Access to opioid agonist treatment (OAT) is limited.
- MAT adherence rates are around 50%.
- Pain is a significant risk factor in OUD.

Weaknesses
- No major weaknesses noted.

2. Investigator(s):
Strengths
- The investigators are very strong and have a history of collaboration.

Weaknesses
- No major weaknesses noted.

3. Innovation:
Strengths
- Development of web-based content targeting pain and comorbid OUD using CBT4CBT-like vignettes is innovative and built on a strong foundation.

Weaknesses
- No major weaknesses noted.

4. Approach:
Strengths
- The team is attentive to reproducibility.
- Plans for the R61 phase are strong and milestones are clearly established.
- The recruitment target for the R33 phase is well within reach based on Liberation enrollment and the pending Bridgeport Clinic.
- Randomization will be accomplished with the computerized urn randomization program.

Weaknesses
- Outcomes will be assessed up to 6-months but not beyond (though it’s worth noting that this was deliberately chosen because of the 3-year duration of the R33 Phase – so this concern is very minor).
- Similar point for lack of comparison to CBT4CBT or COPES alone. This concern is weighted a bit more heavily compared to the outcome duration because there are questions about the suitability of CBT4CBT and COPES without integration in addressing the outcomes in question

(i.e., maybe no combination or augmentation is needed). This concern is diminished by the strong expert and stakeholder engagement in the R61 Phase.

5. Environment:
Strengths
- Yale (and Liberation) is an excellent environment for this work.

Weaknesses
- No major weaknesses noted.

Study Timeline:
Strengths
- Strong, well-defined timelines.

Weaknesses
- No major weaknesses noted.

R33 to R61 Transition Milestones:
Strengths
- Milestones are clear and logically lead to next steps.

Weaknesses
- No major weaknesses noted.

Protections for Human Subjects:
Acceptable Risks and/or Adequate Protections
- Acceptable risks.

Data and Safety Monitoring Plan (Applicable for Clinical Trials Only):
- Acceptable
    - Reasonable plan to monitor risk.

Inclusion Plans:
- Sex/Gender: Distribution justified scientifically
- Race/Ethnicity: Distribution justified scientifically
- For NIH-Defined Phase III trials, Plans for valid design and analysis: Not applicable.
- Inclusion/Exclusion Based on Age: Distribution justified scientifically
- It's a reasonably balanced sample.

Vertebrate Animals:
Not Applicable (No Vertebrate Animals)

Biohazards:
Not Applicable (No Biohazards)

Resource Sharing Plans:
Acceptable
All data are de-identified before sharing, using procedures that are in compliance with HIPPA and Yale Human Investigation Committee standards.

Authentication of Key Biological and/or Chemical Resources:
Not Applicable (No Relevant Resources)

Budget and Period of Support:
Recommend as Requested

CRITIQUE 4

Significance: 4
Investigator(s): 1
Innovation: 1
Approach: 4
Environment: 1

Overall Impact:
R61 Phase: There is a high likelihood that the R61 phase will be successful, as a) the investigators are beginning with two existing programs that have been tested multiple times and b) they are convening panels of clinicians with expertise in treating comorbid OUD and chronic pain, and Liberation (study site) clinicians with expertise in chronic pain; and c) the milestones not only have face validity, but are also supported by the investigators' experience in creating the existing programs.
R33 Phase: Overall, the analytic approach and controls proposed for the primary and secondary outcomes of the R33 phase are appropriate. One weakness of note is that the investigators propose the assessment of differences in healthcare service utilization, and the associated costs, as a secondary outcome, but fail to adequately discuss their analytic plan for these data. It is also unclear whether the investigators plan to assess the costs of implementing and continually managing the program.
Summary: This study will help address a very important question in the treatment of OUD; i.e., can CBT improve opioid agonist therapy (OAT) adherence. However, focusing solely on the population of persons with comorbid chronic pain and OUD reduces generalizability, as the research question is an important one for all persons with OUD. The investigators posit that a CBT program dually focused on chronic pain and OUD treatment with OAT will result in improved outcomes, because existing evidence indicates that pain may reduce the likelihood of "good" OAT outcomes, and CBT is associated with improvements in chronic pain. However, there is little justification that a specialized version of a CBT program for persons with comorbid OUD and chronic pain, who are on OAT, is required. Therefore, aside from the need for more research on whether CBT in general enhances OAT, a better research question may be whether an integrated version performs better than either a CBT for chronic pain, or CBT for OAT alone.

1. Significance:
Strengths
- Roughly 11% of US adults experience chronic pain, and those who are prescribed opioids are at increased risk of developing opioid use disorder.
- Evidence indicates an association between pain and worse outcomes for persons on opioid agonist therapy (OAT).
- Cognitive behavioral treatment (CBT) for chronic pain (CP) has been recommended as a first-line therapy to reduce pain and improve function.
- Technology-based CBT interventions can reduce barriers to in-person CBT treatment; for example, those associated with travel, time (due to frequent sessions), and shortages of trained therapists.
- The study's primary research question is an important one, as there is limited evidence that CBT in conjunction with OAT can improve outcomes; however, many believe it does.

Weaknesses
- Effects of CBT on non-headache pain have typically been in the small-to-moderate range, and most studies have excluded persons with SUD.
- There is limited evidence that persons with comorbid chronic pain and OUD require specialized treatment.
- Focusing solely on persons with chronic pain reduces generalizability, as the research question is an important one for all persons with OUD.

2. Investigator(s):
Strengths
- Drs. Carroll and Heapy have put together a strong team.
- Dr. Carroll developed the CBT manual published by NIDA, and has experience conducting trials testing efficacy of CBT, including among persons with OUD.
- Dr. Carroll developed a computer-based CBT platform, and has successfully tested it in numerous studies.
- Dr. Heapy is highly experienced in developing and testing psychological interventions for chronic pain.

Weaknesses
- No major weaknesses noted.

3. Innovation:
Strengths
- A web-based, integrated intervention targeting chronic pain for persons with OUD on opioid agonist treatment is innovative.
- The daily collection of patient-reported information via interactive voice response (IVR) will allow for examination of the relationships of OAT outcomes and factors such as pain ratings and its fluctuations, craving, sleep problems, and exercise.

Weaknesses
- No major weaknesses noted.

4. Approach:
Strengths
- The scientific approach is rigorous.
- There is a high likelihood that the R61 phase will be successful, as a) the investigators are beginning with two existing programs that have been tested multiple times, b) they are convening panels of clinicians with expertise in treating comorbid OUD and chronic pain, and Liberation (study site) clinicians with expertise in chronic pain; and c) the milestones not only have face validity, but are also supported by the investigators' experience in creating the existing programs.
- Overall, the analytic approach and controls proposed for the primary and secondary outcomes of the R33 phase are appropriate.

Weaknesses
- Assessing differences in healthcare service utilization, and the associated costs, is highlighted as a secondary outcome for the R33 phase; however, there is no actual discussion of how these data will be analyzed.
- It is also unclear whether the investigators plan to assess the costs of implementing and continually managing the program.

5. Environment:
Strengths
- Strong scientific environment.

Weaknesses
- No major weaknesses noted.

Study Timeline:
Strengths
- The study timeline is appropriate.

Weaknesses
- No major weaknesses noted.

R33 to R61 Transition Milestones:
Strengths
- Milestones are appropriate to ensure a successful transition from the R61- to the R33 phase.

Weaknesses
- No major weaknesses noted.

Protections for Human Subjects:
Acceptable Risks and/or Adequate Protections
Data and Safety Monitoring Plan (Applicable for Clinical Trials Only):
- Acceptable

Inclusion Plans:
- Sex/Gender: Distribution justified scientifically.
- Race/Ethnicity: Distribution justified scientifically.
- For NIH-Defined Phase III trials, Plans for valid design and analysis: Not applicable.
- Inclusion/Exclusion Based on Age: Distribution justified scientifically.

Vertebrate Animals:
Not Applicable (No Vertebrate Animals)

Biohazards:
Not Applicable (No Biohazards)

Resource Sharing Plans:
Acceptable

Authentication of Key Biological and/or Chemical Resources:
Not Applicable (No Relevant Resources)

Budget and Period of Support:
Recommend as Requested

CRITIQUE 5

Overall Impact:
The investigators propose to develop, refine, and test a web-based intervention that addresses pain management in patients with OUD. The topic being addressed is quite important clinically. The results of the study could impact current clinical practice since the intervention is potentially disseminable. The principal investigators (Drs. Carroll and Heapy) have extensive complementary expertise in the development and evaluation of novel behavioral therapies and psychological interventions for patients with OUD and chronic pain with or without MAT. There is a track record of collaborations among the collaborators and principal investigators. A multiple principal investigator leadership plan is in place. The study team has the expertise and many years of experience for the clinical trial proposed using a behavioral intervention approach and computer-based training. However, statisticians play key role in clinical trials, particularly those involving behavioral intervention and survey instruments. It is not clear if there is enough expertise in biostatistics for the project although Ms. Charla Nich, a master level statistician working with the principal investigators for many years over several projects was mentioned in the application. Her biographical sketch, however, should have been included. The academic environment is strong. The studies proposed are novel and have many positive features. The

availability of potentially eligible patients with OUD and chronic pain treated with MAT was noted. Daily monitoring of pain and its fluctuation using interactive voice response is thorough. Detailed measurements in 3 components were provided: integrated (5A's adherence), COPES (behavioral) and CBT4CBT (craving, change of thoughts and decision making). A reasonable sample size estimation and analytic plan was given, especially for some of the exploratory aims.
R61 Phase: The sequence of proposed studies will enable the investigators to further develop and refine the novel intervention called CBT4CBT. The sequence is logical with one activity building on the prior one. Well described core principles will guide intervention development and multiple stakeholders will provide input and feedback. The milestones provided are achievable. The feasibility trial proposed is very small (n = 5) to inform the larger clinical trial.
R33 Phase: The randomized clinical trial is well designed. A detailed plan for monitoring and handling clinical deterioration is included. The investigators have the experience using computer-based training and cognitive behavioral therapy (CBT) in patients with OUD and chronic pain treated with MAT. Strong data of previous work from the group of investigators to deliver the preliminary data in the R61 phase, i.e. developing the integrated CBT4CBT cooperative pain education and self-management (COPES) program and generating pilot data using the program is provided. The study uses a web-based data collection system, which is a strength. As retention and adherence are measured longitudinally, the statistical analysis plan (e.g. logistic regression and MANOVAs) does not fully reflect on that. The study lacks appropriate considerations for issues of false discovery rate as there are multiple tests and comparisons for several outcomes (e.g. adherence and pain interference) and lacks details of multilevel models for exploratory analysis. In addition, as some of the 160 patients to be enrolled in the R33 phase will be enrolled in year 4, some of the patients won't have 9 months of follow-up (as required by Specific Aim 2) by month 55 based on the study timeline put in the application.
Summary: This is a well written application that tests a novel web-based intervention. Its results could have an impact on clinical practice in this field. Although some concerns including statistical considerations and timeline are noted, it is felt that these could be easily addressed and that meaningful findings could thereby be achieved.

THE FOLLOWING SECTIONS WERE PREPARED BY THE SCIENTIFIC REVIEW OFFICER TO SUMMARIZE THE OUTCOME OF DISCUSSIONS OF THE REVIEW COMMITTEE, OR REVIEWERS' WRITTEN CRITIQUES, ON THE FOLLOWING ISSUES:

PROTECTION OF HUMAN SUBJECTS: ACCEPTABLE

INCLUSION OF WOMEN PLAN: ACCEPTABLE

INCLUSION OF MINORITIES PLAN: ACCEPTABLE

INCLUSION OF CHILDREN PLAN: ACCEPTABLE

COMMITTEE BUDGET RECOMMENDATIONS: The budget was recommended as requested.

Footnotes for 1 R61 AT010619-01; PI Name: CARROLL, KATHLEEN M.

NIH has modified its policy regarding the receipt of resubmissions (amended applications). See Guide Notice NOT-OD-14-074 at http://grants.nih.gov/grants/guide/notice-files/NOT-OD-14-074.html. The impact/priority score is calculated after discussion of an application by averaging the overall scores (1-9) given by all voting reviewers on the committee and

multiplying by 10. The criterion scores are submitted prior to the meeting by the individual reviewers assigned to an application, and are not discussed specifically at the review meeting or calculated into the overall impact score. Some applications also receive a percentile ranking. For details on the review process, see http://grants.nih.gov/grants/peer_review_process.htm#scoring.

MEETING ROSTER

National Center for Complementary and Integrative Health Special Emphasis Panel
NATIONAL CENTER FOR COMPLEMENTARY & INTEGRATIVE HEALTH
HEAL Initiative: Behavioral and Social Interventions to Improve Adherence to Medication Assisted Treatment for Opioid Use Disorders
ZAT1 SM (48)
06/21/2019

Notice of NIH Policy to All Applicants: Meeting rosters are provided for information purposes only. Applicant investigators and institutional officials must not communicate directly with study section members about an application before or after the review. Failure to observe this policy will create a serious breach of integrity in the peer review process, and may lead to actions outlined in NOT-OD-14-073 at https://grants.nih.gov/grants/guide/notice-files/NOT-OD-14-073.html and NOT-OD-15-106 at https://grants.nih.gov/grants/guide/notice-files/NOT-OD-15-106.html, including removal of the application from immediate review.

CHAIRPERSON(S)

RUSH, CRAIG R, PHD
PROFESSOR
DEPARTMENT OF BEHAVIORAL
SCIENCE AND PSYCHIATRY
COLLEGE OF MEDICINE
UNIVERSITY OF KENTUCKY
LEXINGTON, KY 40536

MEMBERS

ALESSI, SHEILA MARIE, PHD
ASSOCIATE PROFESSOR
DEPARTMENT OF PSYCHIATRY
CALHOUN CARDIOLOGY CENTER
UNIVERSITY OF CONNECTICUT HEALTH CENTER
FARMINGTON, CT 06030

BLONDELL, RICHARD D, MD
PROFESSOR
DEPARTMENT OF FAMILY MEDICINE
STATE UNIVERSITY OF NEW YORK
UNIVERSITY AT BUFFALO
BUFFALO, NY 14215

CARREIRO, STEPHANIE P, MD
ASSISTANT PROFESSOR
DIVISION OF TOXICOLOGY
DEPARTMENT OF EMERGENCY MEDICINE
UNIVERSITY OF MASSACHUSETTS MEDICAL SCHOOL
55 LAKE AVENUE NORTH
WORCESTER, MA 01655

FEASTER, DANIEL J, PHD
PROFESSOR
DIVISION OF BIOSTATISTICS
DEPARTMENT OF PUBLIC HEALTH SCIENCES
MILLER SCHOOL OF MEDICINE
UNIVERSITY OF MIAMI
MIAMI, FL 33136

FIELD, CRAIG A, PHD
ASSOCIATE PROFESSOR
DEPARTMENT OF PSYCHOLOGY
COLLEGE OF LIBERAL ARTS
THE UNIVERSITY OF TEXAS AT EL PASO
EL PASO, TX 79912

FRESCO, DAVID M, PHD
PROFESSOR
DEPARTMENT OF PSYCHOLOGICAL SCIENCES
KENT STATE UNIVERSITY
KENT, OH 44242

FRIEDMANN, PETER D, MD
ASSOCIATE DEAN FOR RESEARCH
OFFICE OF RESEARCH
UNIVERSITY OF MASSACHUSETTS
MEDICAL SCHOOL - BAYSTATE
SPRINGFIELD, MA 01199

FU, PINGFU, PHD
ASSOCIATE PROFESSOR
DEPARTMENT OF POPULATION AND
QUANTATIVE HEALTH SCIENCES
CASE WESTERN RESERVE UNIVERSITY
CLEVELAND, OH 44106

GORDON, ADAM JOSEPH, MD
PROFESSOR
DEPARTMENT OF MEDICINE AND PSYCHIATRY
SCHOOL OF MEDICINE
UNIVERSITY OF UTAH
SALT LAKE CITY, UT 90315

GUSTAFSON, DAVID H, PHD
RESEARCH PROFESSOR AND DIRECTOR
CENTER FOR HEALTH ENHANCEMENT SYSTEM STUDIES
COLLEGE OF ENGINEERING
UNIVERSITY OF WISCONSIN, MADISON
MADISON, WI 53706

JARLENSKI, MARIAN PATRICIA, PHD
ASSISTANT PROFESSOR
DEPARTMENT OF HEALTH POLICY
AND MANAGEMENT
UNIVERSITY OF PITTSBURGH
GRADUATE SCHOOL OF PUBLIC HEALTH
PITTSBURGH, PA 15261

KEEFE, FRANCIS J., PHD
PROFESSOR
DEPARTMENT OF MEDICINE
DIVISION OF RHEUMATOLOGY AND IMMUNOLOGY
DUKE UNIVERSITY SCHOOL OF MEDICINE
DURHAM, NC 27705

KENDZOR, DARLA ELIZABETH, PHD
ASSOCIATE PROFESSOR
DEPARTMENT OF FAMILY AND PREVENTIVE MEDICINE
OKLAHOMA TOBACCO RESEARCH CENTER
UNIVERSITY OF OKLAHOMA HEALTH SCIENCES CENTER
OKLAHOMA CITY, OK 73104

KIDORF, MICHAEL S, PHD
ASSOCIATE PROFESSOR
DEPARTMENT OF BEHAVIORAL BIOLOGY
ASSOCIATE DIRECTOR
ADDICTION TREATMENT SERVICES
JOHNS HOPKINS BAYVIEW MEDICAL CENTER
BALTIMORE, MD 21224-6823

LATKIN, CARL A, PHD
PROFESSOR
DEPARTMENT OF HEALTH POLICY AND MANAGEMENT
SCHOOL OF PUBLIC HEALTH
JOHN HOPKINS UNIVERSITY
BALTIMORE, MD 21205

LENZE, ERIC J, MD, PHD
PROFESSOR
DEPARTMENT OF PSYCHIATRY
WASHINGTON UNIVERSITY SCHOOL OF MEDICINE
SAINT LOUIS, MO 63110

LI, GUOHUA, DRPH, MD
PROFESSOR AND DIRECTOR
COLUMBIA UNIVERSITY
NEW YORK CITY, NY 10032

LINAS, BENJAMIN P., MD
ASSOCIATE PROFESSOR
DEPARTMENT OF MEDICINE
BOSTON UNIVERSITY SCHOOL OF MEDICINE
BOSTON, MA 02118

LORD, SARAH E, PHD
ASSISTANT PROFESSOR AND DIRECTOR
DISSEMINATION AND IMPLEMENTATION CORE
CENTER FOR TECHNOLOGY AND BEHAVIORAL HEALTH
DARTMOUTH PSYCHIATRIC RESEARCH CENTER
GEISEL SCHOOL OF MEDICINE AT DARTMOUTH COLLEGE
LEBANON, NH 03766

LOVEJOY, TRAVIS IAN, PHD
ASSOCIATE PROFESSOR OF PSYCHIATRY
DEPARTMENT OF PSYCHIATRY
SCHOOL OF MEDICINE
OREGON HEALTH AND SCIENCE UNIVERSITY
PORTLAND, OR 97239

MCGEARY, DONALD DOUGLAS, PHD
ASSOCIATE PROFESSOR
DEPARTMENT OF PSYCHIATRY
SCHOOL OF MEDICINE
UNIVERSITY OF TEXAS HEALTH SCIENCE CENTER
SAN ANTONIO, TX 78229

MCHUGH, REBECCA KATHRYN, PHD
ASSISTANT PROFESSOR
DEPARTMENT OF PSYCHIATRY
HARVARD MEDICAL SCHOOL
DIVISION OF ALCOHOL AND DRUG ABUSE
MCLEAN HOSPITAL
BELMONT, MA 02478

MURPHY, SEAN M., PHD
ASSOCIATE PROFESSOR
DEPARTMENT OF HEALTHCARE POLICY
AND RESEARCH
WEILL CORNELL COLLEGE OF MEDICINE
NEW YORK, NY 10065

SINDELAR, JODY L., PHD
PROFESSOR
DEPARTMENT OF HEALTH POLICY AND MANAGEMENT
YALE UNIVERSITY SCHOOL OF PUBLIC HEALTH
NEW HAVEN, CT 06520-8034

SINHA, RAJITA, PHD
PROFESSOR
CHIEF, PSYCHOLOGY SECTION IN PSYCHIATRY
DIRECTOR, YALE INTERDISCIPLINARY STRESS CENTER
YALE UNIVERSITY SCHOOL OF MEDICINE
NEW HAVEN, CT 06519-1717

TOMPKINS, DAVID ANDREW, MD
DIRECTOR
DIVISION OF SUBSTANCE ABUSE AND ADDICTION MEDICINE
ZUCKERBERG SAN FRANCISCO GENERAL HOSPITAL
ASSOCIATE CLINICAL PROFESSOR, DEPART OF
PSYCHIATRY
UNIVERSITY OF CALIFORNIA SAN FRANCISCO
SAN FRANCISCO, CA 94110

UEBELACKER, LISA A, PHD
ASSOCIATE PROFESSOR
DEPARTMENT OF PSYCHIATRY AND HUMAN BEHAVIOR
BUTLER HOSPITAL
BROWN UNIVERSITY
PROVIDENCE, RI 02906

WANG, ZHU, PHD
ASSOCIATE PROFESSOR
DEPARTMENT OF EPIDEMIOLOGY & BIOSTATISTICS
UT HEALTH SAN ANTONIO
SAN ANTONIO, TX 78229

WATSON, DENNIS PAUL, PHD
ASSOCIATE PROFESSOR
CENTER FOR DISSEMINATION & IMPLEMENTATION SCIENCE
DEPARTMENT OF MEDICINE
COLLEGE OF MEDICINE
UNIVERSITY OF ILLINOIS AT CHICAGO
INDIANAPOLIS, IN 46202

WINSTANLEY, ERIN L, PHD
ASSOCIATE PROFESSOR
DEPARTMENT OF BEHAVIORAL MEDICINE AND PSYCHIATRY
SCHOOL OF PHARMACY
WEST VIRGINIA UNIVERSITY
MORGANTOWN, WV 26506

WOODY, GEORGE EDWARD, MD
PROFESSOR
DEPARTMENT OF PSYCHIATRY
PERELMAN SCHOOL OF MEDICINE
UNIVERSITY OF PENNSYLVANIA
PHILADELPHIA, PA 19106

YARBOROUGH, BOBBIJO H., PSYD
INVESTIGATOR
KAISER PERMANENTE NORTHWEST
CENTER FOR HEALTH RESEARCH
PORTLAND, OR 97227

SCIENTIFIC REVIEW OFFICER

NANESCU, SONIA ELENA, PHD
SCIENTIFIC REVIEW ASSISTANT [C]
OFFICE OF SCIENTIFIC REVIEW
DIVISION OF EXTRAMURAL ACTIVITIES
NCCIH, NIH
6707 DEMOCRACY BLVD., SUITE 401
BETHESDA, MD 20892

SCHMIDT, MARTINA, PHD
CHIEF
OFFICE OF SCIENTIFIC REVIEW
NATIONAL CENTER FOR COMPLEMENTARY
AND INTEGRATIVE HEALTH
NATIONAL INSTITUTES OF HEALTH
BETHESDA, MD 20892

EXTRAMURAL SUPPORT ASSISTANT

ABUSAKRAN, HAJER KHALIL
EXTRAMURAL SUPPORT ASSISTANT
OFFICE OF SCIENTIFIC REVIEW
DIVISION OF EXTRAMURAL ACTIVITIES
NATIONAL CENTER FOR COMPLEMENTARY AND
INTEGRATIVE
HEALTH, NIH
BETHESDA, MD 20892-5475

Consultants are required to absent themselves from the room during the review of any application if their presence would constitute or appear to constitute a conflict of interest.
